# A Genetic Approach to the Development of New Therapeutic Phages to Fight *Pseudomonas Aeruginosa* in Wound Infections

**DOI:** 10.3390/v5010015

**Published:** 2012-12-21

**Authors:** Victor Krylov, Olga Shaburova, Sergey Krylov, Elena Pleteneva

**Affiliations:** Laboratory for Bacteriophages Genetics. Mechnikov Research Institute of Vaccines and Sera, RAMS, 5a, Maliy Kazenniy per., Moscow, 105064 Russia; E-Mails: oshabs@mail.ru (O.S.); sergeykrylovv@gmail.com (S.K.); f2600@yandex.ru (E.P.)

**Keywords:** *Pseudomonas aeruginosa* bacteriophages diversity, noncanonical relations of phages and bacteria, phage’s migrations, phage genomes instability, pseudolysogeny and pseudovirulence, phage therapy as a kind of personalized medicine

## Abstract

*Pseudomonas aeruginosa* is a frequent participant in wound infections. Emergence of multiple antibiotic resistant strains has created significant problems in the treatment of infected wounds. Phage therapy (PT) has been proposed as a possible alternative approach. Infected wounds are the perfect place for PT applications, since the basic condition for PT is ensured; namely, the direct contact of bacteria and their viruses. Plenty of virulent (“lytic”) and temperate (“lysogenic”) bacteriophages are known in *P. aeruginosa.* However, the number of virulent phage species acceptable for PT and their mutability are limited. Besides, there are different deviations in the behavior of virulent (and temperate) phages from their expected canonical models of development. We consider some examples of non-canonical phage-bacterium interactions and the possibility of their use in PT. In addition, some optimal approaches to the development of phage therapy will be discussed from the point of view of a biologist, considering the danger of phage-assisted horizontal gene transfer (HGT), and from the point of view of a surgeon who has accepted the Hippocrates Oath to cure patients by all possible means. It is also time now to discuss the possible approaches in international cooperation for the development of PT. We think it would be advantageous to make phage therapy a kind of personalized medicine.

## 1. Introduction

Gram-negative bacteria of species *Pseudomonas aeruginosa* may be found in different natural habitats, because they easily adapt to different conditions. The capability for quick adaptation is the main reason that identifies them as opportunistic pathogens. They cause infections in immune compromised patients or patients with cystic fibrosis—a frequently occurring hereditary disease in Caucasians. *P. aeruginosa* strains are common components in microbial communities of different origins. They have acquired the status of hospital pathogens, and may be isolated from clinical samples taken from the wounds, sputum, bladder, urethra, vagina, ears, eyes and respiratory tract. The emergence of resistance to the most powerful new antibiotics in such clinical *P. aeruginosa *strains, occurring even during treatment [1,2,3,4,5,6,7,8,9], makes the fight with *P. aeruginosa* hospital pathogens a great problem.

**Figure 1:**
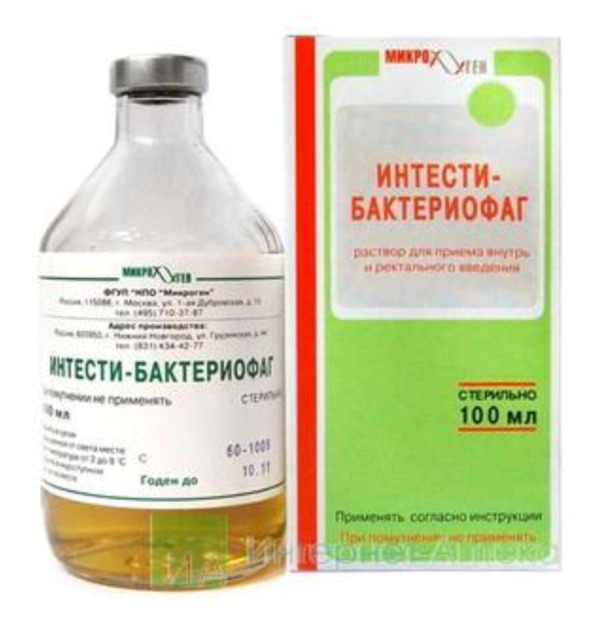
Commercially produced mix of phages “Intesti” (ImBio Nizhny Novgorod, Russia).

Genomes in most hospital strains of *P. aeruginosa* contain pathogenic islands, where genes, coding many factors of pathogenicity and virulence of this bacterial species such as phospholipase C elastase, protease, siderophore, DNAse, pyocyanin *etc*., are located simultaneously with genes controlling multiple drug resistance. One of these large genomic islands, PAPI-1, can be transformed into an extrachromosomal circular form, plasmid, after precise excision from a bacterial chromosome [10]. Sometimes such plasmid acquires the ability to be transferred into other recipient *P. aeruginosa* strains by a conjugative mechanism, via a type IV pilus [11]. Such migration quickly disseminates an antibiotic resistance into new strains, thus making the use of antibiotics useless. This is the reason for a quite unexpected renaissance of phage therapy, the use of bacterial viruses in the treatment of bacterial infections, which was proposed by Felix D’Herelle in 1917, immediately after the finding of bacteriophages [12]. The bacteriophage treatment was applied in medical practice with varying success until the introduction of antibiotics; then, because of the great success and simplicity in use of antibiotics, phages were no longer regarded as a serious tool in anti-infective therapy. However, in Russia, Poland and Georgia the use of phage therapy has not ceased and continues to the present day. Given the frequent epidemics of food borne diseases and the increase of many enteric pathogens resistant to antibiotics [13,14,15,16,17], it is useful to remember that some specific phage compositions introduced by F. D’Herelle (but permanently updated) are used with success in the treatment and in the prevention of intestinal infections (see Figure 1).

In Eastern Europe, bacteriophages are widely used in surgical wards [18,19,20]. Apparently, this trend in phage therapy of *P. aeruginosa* caused infections will be used further, considering the failure of the development of safe vaccines against *P. aeruginosa* [21,22,23].

Bacteriophages were an excellent model of genetic research. Many of the basic concepts of modern biology and the different elements of the methodology in biological and medical sciences have emerged as a result of studying the genetics of phages from the 1940s to 1970s. Being used in other areas, they have become a powerful boost to research of various pro- and eukaryotic systems. In the last two decades, interest in bacteriophages increased significantly again. One reason is the above mentioned occurrences of multiple antibiotic resistant bacteria and the hope that the use of live phages or their products—structured or molecular bacteriocins—antibacterial peptide phage origin—can help in the treatment of bacterial infections. In our opinion, there is another, no less significant reason for a detailed study of bacteriophages. As it has turned out, bacteriophages (both temperate and virulent) are actively involved in the evolution of bacteria, including pathogens, accomplished through different kinds of transduction (general and specialized) horizontal gene transfer (HGT). Studies of the structure of pathogenic islands of different bacterial species confirmed the presence in them of complete or fragmented genomes of temperate phages. Many of the new forms of infective diseases (so-called “emerging diseases”) are caused by the appearance in pathogenic islands of previously known pathogens of new genes introduced by phages, plasmids, and transposons (which can be considered as largely related genetic elements that form a common pool of evolutionary active genes). The increased interest in the use of bacteriophages as a replacement for antibiotics in the West has led to an increase in the number of publications illustrating the possibility of actual use of phages in human and veterinary medicine, in finding and describing new phages. Here are the numerous review papers related somehow to bacteriophages that were published recently [24,25,26,27,28,29,30]. Extremely bold proposals have been made of the possible use of bacteriophages as antiseptic additives in ready to eat foods, which may potentially be infected with pathogenic bacteria. There are ideas for use of bacteriophages and their products to retard spoilage of foodstuffs during the time of storage or for the prevention of endemic infections and food poisoning associated with the consumption of potentially contaminated food (phage preparations of the «Intesti» type) (see above). The need to quickly find, some alternative to antibiotics is evident. However, in the case of phages there is an evident problem: how to eliminate the possible undesirable activity of phages with the emergence of new pathogens in the process of introducing phage therapy. Some other problems must also be solved if there is an intention to raise the level of phage therapy up to accepted standards in modern medicine. Among such problems are ensuring reproducible results of phage therapy, its long-term use and achieving the required standards for therapeutic phages and their mixtures. Can the introduction of phage therapy be a cause of the emergence of pathogens with new properties? There are also some questions about the prerequisite of high level cooperation, including a quick exchange with bacteriophages capable of stopping infectional outbreaks. We consider some of these issues later, but first we discuss the natural limitations in the use of bacteriophages that distinguish them from antibiotics. This assesses the real prospects of live phage therapy and also helps understand why phage therapy is not the main procedure of treatment for some diseases in the countries where its use was not interrupted after introduction of antibiotics.

## 2. The Goal, Objectives and Content of the Present Review

Many studies related in one way or another to bacteriophages have been published in the last 10–15 years. They concern, the numerous advantages of phage therapy and specific features of a large number of newly isolated phages have been discussed. The list of phages with sequenced and annotated genomes is in constant enlargement in NCBI.

Currently, there has been established a certain unofficial algorithm in the studies of newly isolated bacteriophages: the isolation of the phage itself, extraction of genomic nucleic acid (in most cases, DNA), electron microscopy of phage particles for classification purposes, genome sequencing and annotation, estimation of evolutionary relationships with other phages and prospects for practical use. Previously initiated as well as new companies are in action, producing and advertising their numerous commercial phage mixtures; newly organized specialized therapeutic bacteriophage treatment centers are operating. We have not provided links to these activities: all this can be found on the Internet.

The purpose of this review is to draw attention to the need for a close examination of the possible features in the behavior of different types of bacteriophages in their actual application, which can be found on the stage of laboratory studies [31], in order to understand the possible consequences and the necessary modifications of the bacteriophages and the methods of their use. The system of phage therapy is fundamentally different from the organization of antibiotic therapy. In supporting the use of bacteriophages in medical practice, we want first of all to justify the need for the introduction of such a system. We start by looking at the natural limitations in the use of phages as therapeutic agents.

## 3. Limitations in the Use of Live Bacteriophages in a Treatment

(1) Phage therapy with living phages is applicable only in cases where it is possible to provide direct contact with the virus and bacteria. However, the direct introduction of even highly purified phage particles in the blood, such as in the case of septicemia (although there are described unique cases of this kind) [32] is very risky. The view that oral administration of phages may cause them to penetrate into the bloodstream, and then into the urine (the concept of “phages are absorbed into blood and cure”), is not justified by rigorous research (although such cases have also been described in the literature) [33]. Our own laboratory experiments did not confirm this. Requirements for direct contact limits the use of phage therapy with wound infections (other than pressure ulcers), urogenital infections, intestinal infections, eye infections and infections of the ear, nose and throat organs. Cystic fibrosis, where bacteria of the species P. aeruginosa are in contact with different phages cannot yet be attributed to the list of infections that are acceptable for phage therapy, although research in this area (in a mouse model) continues [34].

(2) Another reason for the limited use of phage therapy is the inability to provide long-term effectiveness of a particular mixture of therapeutic phages. In the manufacturing companies, producing commercial phage mixtures, it is generally accepted that included in such products are phages with the broadest spectrum of lytic activity for each bacterial species involved. However, regardless of the spectrum of lytic activity of phages in the mixtures, bacterial mutants of a species that reveal resistance to all phages in the mixture may arise even after a few days after the start of treatment. Thus, a single mutation in the genome of Gram-negative bacteria such as *P. aeruginosa*, leading to resistance to adsorption, may prevent the growth and lytic activity several unrelated phages [35]. It is known from personal contact with surgeons of departments of purulent infections, that phage-resistant clones often arise after short time use of polyvalent phage mixtures. Such resistant clones can then replace the previous hospital pathogens. We consider the properties of these pathogens and their relation to phages later. Usually renewal of phages in commercial mixtures is quite rare, a few times a year. Therefore, the rapid accumulation of phage-resistant mutants can lead very quickly to loss of activity of the applied phage mixture. The only possible solution to the problem is the replacement of such an ineffective phage mixture with the mixture from another manufacturer, in the hope that the phages in the new mixture have a different spectrum of lytic activity. This is a temporary albeit not very reliable solution.

(3) The obvious aim of each producer of therapeutic phages is to create a preparation with the highest range of final lytic activity, which can be used without replacement for as long as possible. Therefore, since the time of F. D’Herelle, in order to ensure maximal activity of therapeutic phages against pathogenic strains, phages for therapy have been produced with the use of pathogenic clinical isolates. Another important reason for such use of clinical (pathogenic) isolates is that it is not always possible to find appropriate non-pathogenic variants with sensitivity to a particular phage, which must be incorporated into the therapeutic mix. This is the positive side of the use in the batch phage production of clinical isolates as hosts. On the other hand, the real pathogen used as a production strain, even when non-lysogenic, obviously contains genes with harmful effects. As a result, phages obtained with the use of such a pathogen, when introduced into the therapeutic mixture, may in the course of treatment, with any kind of transduction, transfer such genes into clinical strains with the resulting emergence of bacteria that cause the disease with symptoms of “emerging diseases”. The only solution is to use only well studied bacterial hosts. In principal, it is necessary to create production strains that are sensitive to different phages, based on well-studied model strains that do not contain dangerous genes in their genomes.

(4) Some weaknesses in planning and use of phage therapy also hinder the successful introduction as a standard treatment or preventive procedure. There is a need to create a well thought out organization at all levels from the isolation of a pathogenic strain to the fast selection of the best phage for treatment. The creation of such an organization requires support on an inter-state level, because its development goes beyond the possibilities of individual researchers or research laboratories, and apparently requires coordination on a number of different levels. The existence of such a system could play a positive role in quick reactions to the occurrence of local epidemics, such as the emergence of the outbreak caused by an enterohemorrhagic (EHEC) strain of *E. coli* serotype O104: H4 in early May 2011 in northern Germany, or in the case of the outbreak of listeriosis in September and October 2011 in the U.S.A. In the U.S.A., for instance, there is a phage mixture active against *Listeria *(integrated drug ListShield™; [36]) which is permitted by the US Food and Drug Administration for use in «ready-to-eat» products. It is possible that the timely distribution of therapeutic phage mixtures amongst the population in areas threatened by epidemiological characteristics could reduce the number of subsequent cases. In the same hypothetical system of organization of phage therapy should be included laboratories involved in research of phages for therapy as well as international collections of bacteriophages [37]. An optimal situation is the case when only such phages active against local pathogens, but not polyvalent mixtures are used in clinics. It is good in the case when there is the possibility to choose such phages *in situ*, in the hospital, and then treatment must be accompanied by constant monitoring to check for arising phage‑resistant variants. The choice of inappropriate phages and use of polyvalent mixtures without checking for the effectiveness of their component phages against particular wound pathogens will affect the efficiency of treatment and, as a consequence, will discredit the idea of phage therapy among doctors and patients.

Therapeutic phages, which are intended for personal use, should definitely go through the standard procedure of classification up to species. This significantly lowers the probability of HGT, revealing the presence in the genomes of phages containing any features that pose a potential danger. Perhaps, phage therapy at some future time could be included into the arsenal of personalized medicine.

## 4. Non-Canonical Interaction of Phages and Bacteria

The original idea of phage therapy is based on the assumption that interactions of phages and bacteria are similar with predator-prey relationships and the development of virulent phage proceeds within the framework of the lytic cycle. This ideal (“canonical”) lytic cycle is as follows: adsorption of the phage to the surface cell structures (adsorption receptors specific for each type of phage); injection of the phage genome into the cell (using the natural pores or after local melting of the cell wall by a lytic enzyme incorporated into the structure of the tail of the phage particle); the implementation of phage development programs written in phage genome; the destruction of the cell envelope from inside by specific phage enzymes and release progeny phage particles; readiness to repeat the same cycle. The ability of cells to divide normally disappears soon after the injection of the phage genome.

In the case of infection cells with temperate phage there can be two outcomes: a typical lytic cycle or lysogenization of the infected cell with a phage genome in prophage condition; prophage can be integrated into the bacterial chromosome or be in a state of plasmid, whose division and distribution in each of the daughter bacterial cells are strictly coordinated with the division of bacteria and replication of bacterial chromosome. A stable state of bacterial cell with prophage is supported through the blockade of lytic genes expression in prophage with a special protein repressor. Sometimes the repressor undergoes inactivation, and the phage starts the lytic cycle of development. However, under certain conditions, there is a deviation from such a course of events that could significantly change the nature of the phage behavior and, as a consequence, the final result of phage therapy. These deviations can result in a pseudolysogenic state of infected bacterial cells. Developmental disorders in the case of temperate phages can prevent the establishment of a lysogenic state (pseudovirulent state).

A good example of the lytic cycle is the development of phage T4. As a model for temperate phage, the development of bacteriophage lambda can be accepted. Nevertheless, it is necessary to recognize that the properties, even of these models, are still far from being fully explored. For example, the functions of 20 proteins coded by the lambda genome were not possible to assign in comparison with the known database. Their functions were studied with specially designed experiments [38]. Authors predicted functions for 12 of the mentioned proteins. So even in the case of phage for which careful research has lasted several decades, the functions of many of the genes are still unknown.

## 5. Pseudolysogeny and Pseudovirulence

### 5.1. Pseudolysogenic Conditions

#### 5.1.1. Some Examples of Reasons for Pseudolysogenic Conditions

Studies of bacteriophages in laboratories are usually accomplished under conditions which are optimal for bacterial host growth. However, interactions of bacteria and phages at the place of infection may occur in different situations. To check their influence on deviations in the canonical interaction of phage and bacteria before introduction of a phage into a therapeutic mixture, it is necessary to conduct special studies under different conditions. The importance of such studies is confirmed by the existence of particular types of phage development, which differ from the canonical lytic and temperate infections. We do not mention here specific bacterial viruses with chronic type infection as their use in therapy or occasional occurrence in phage therapeutic mixtures in our opinion may be very dangerous [37]. Let us now consider some different examples of pseudolysogeny:

(a) Frequent reasons for establishment of pseudolysogeny and its maintenance are changes of cultural conditions in the background of specific bacterial and phage genotypes. Typical results were obtained in the study of *P. aeruginosa*, slowly growing in the presence of its phage in a chemostat continuous culture model. The frequency of pseudolysogens increased as cells were starved. According to the authors, pseudolysogeny is specific phage strategy to survive in periods of starvation of their hosts [39].
(b) Transition of lysogenic cells from active multiplication into stationary phase can also stimulate high frequency of phage loss, as has been found in the study of the gram-positive bacteria *Propionibacterium acnes* with a group of closely related temperate inducible phages. Bacterial clones surviving such an event were susceptible to new infection by the same phages. Most likely, the phage genome in this case was not integrated into the bacterial chromosome [40].
(c) Bacteria may acquire a capability for lysogeny as result of mutation. Phage VHS1 produces clear plaques on its host *Vibrio harveyi* (strain VH1114). Bacterial clones have been isolated which could be lysogenized but being lysogens they produced cured cells after approaching the stationary growth phase. These bacteria had inherited differences proving their mutational origin [41].
(d) Sometimes pseudolysogeny is a result of several different genetical modifications in phage genome and/or in a bacterial genome. Neurotoxins C and D of *Clostridium botulinum* are encoded by genes of bacteriophages. The genome of converting phage c-st was sequenced and annotated. As it turned out, c-st prophage is present as a circular plasmid. Plasmids (and plasmid prophages) code special proteins, necessary to resolve plasmid multimer partition, and segregation, but it is possible that their activity is not sufficient to ensure the stable inheritance of c-st as plasmid. Besides, there is another important reason for the plasmid instability. A remarkable feature of the c-st genome is the abundance of IS elements (altogether 12 copies). The presence of such a number of IS is unexpected for a viable phage. The authors suggest that possible recombination between these multiple elements is the basic reason for the plasmid prophage instability. This case of pseudolysogeny may be considered as a typically weakened impaired lysogenic state [42].

Detailed studies of the regulation systems of the temperate phage lambda *E. coli* demonstrated the dependence of the stability of the lysogenic state of functioning of a group of genes that affect the expression of the gene encoding the repressor. However, apparently with new phages allocated from the natural environment there are many cases that cannot be explained by reference to known patterns. 

#### 5.1.2. Pseudolysogeny and Therapeutic Phages for *P. aeruginosa*: General Considerations

Our laboratory, for a number of years, has studied different bacteriophages, active in pseudomonades of different species. In the course of such work, we have met some situations which evidenced the “non‑canonical” behavior of phages and phage-sensitive bacteria for *P. aeruginosa.* In relation to phage therapy, it may be of interest to consider some cases of non-canonical interactions for phages from existing commercial mixtures. Besides, it is necessary to take into account the special role of temperate phages in horizontal genetic transfer. Such observations can have certain practical significance and can be taken into consideration to enhance phage therapy of infected wounds or hospital infections. The studies of non-canonical interactions of phages and bacteria are intended primarily to assess the possibility and probability of occurrence during the treatment of hybrid phages capable of transposition, conversion, and lysogenization, *i.e.*, participating in the process of HGT. The need for such studies has been proved because even the sequencing and annotation of the genome cannot always predict the behavior of bacteriophages in the real world. Similarly, *in silico* experiments on the interaction of controlled phage proteins cannot confirm the safety of their functions or prove the significance of the function of these proteins for the viability or lytic activity of the phage. In relation to *P. aeruginosa* caused infections, there are detailed studies of genomes of several phage groups which may be considered as favorites for phage therapy, such as KMV-like, PB1-like, N4-like and phiKZ-like phages, which are be found in some commercial mixtures. Different phages of these four species are the frequent sources used to replenish commercial mixtures. It has been assumed that the representatives of individual phages belonging to certain species are not very different from each other. However, it is impossible to suggest the identity of the behavior of all phages of the same species without their detailed comparison. Certainly, there are some limitations in the attempts for immediate applications of results obtained in the laboratory (“Petri dish studies”) applied to conditions existing in a real infected wound. Nevertheless the basic processes in relation of *P. aruginosa* and its bacteriophages in the case of surface infections reveal a good similarity in both cases (biofilm formation, adhesion, alginate production, basic features of phage infections as transpositions, plasmid and phage migrations, *etc*.). We consider here several different deviations from canonical interactions of phages and *P. aeruginosa*, which may manifest themselves in the infected wound. In addition, we discuss the possible use of temperate phages in therapy and the influence of wound bacterial *P. aeruginosa* strains on phage growth. Some *P. aeruginosa* temperate transducing phages ((B3, G101, F116) [43] reveal a natural ability to infect strains as *Burkholderia cepacia* which may be considered a proof of common origin of the species [44].

#### 5.1.3. Pseudolysogeny in the Case of phiKZ-Like Phages

PhiKZ (access number NC_004629) is a very special giant phage, which has the specific structure of capsid with a spiral inner body [45,46]. The phiKZ-like phages active on *P. aeruginosa* were classified into three species (phiKZ, Lin68, EL) [47,48]. Different phiKZ-lke phages are permanent components of commercial phage therapeutic polivalent mixtures. One of the attractive features of this phage species when used in commercial preparations is its capability to produce high final yields. The phiKZ-like phages are a very common component of phage biota in water and soil, and may be isolated in different regions [49]. PhiKZ was found to contain several genes coding for orthologs of proteins of pathogenic prokaryotes, unrelated phages, and some eukaryotes [50]. It is not known up to now, whether these genes belong to the phage genome and code for proteins essential for phage development or whether they represent “genetic noise”, captured by the phage during migration and potentially playing a detrimental role.

These issues are of interest for several reasons. First, commercial mixtures used in phage therapy sometimes show a regional specificity. However, phages of species phiKZ, isolated from geographically distant regions, have just small genetic differences from phage phiKZ. Each of the phages has a quite broad (more than 20%) lytic activity spectrum. Such phages are extremely useful to kill specific bacterial strains resistant to other phages. We have found that the drastic differences in interactions of phiKZ-like phages with their host are dependent on the multiplicity of infection (m.o.i.). In a one step growth cycle experiment (m.o.i. in a range of 1–5 particles), phiKZ behaves as a typical virulent phage. All infected cells were killed with liberation of a not very high number of phage particles [45]. However, there was a significant increase of m.o.i. of sensitive bacteria with phiKZ-like phages, which arises when they are grown in biofilm conditions. As a result, these phages produce huge amounts of progeny particles, which exceed phage concentrations many times in the case of low m.o.i. In the genomes of all phiKZ-like phages that have been sequenced up to now, no gene coding DNA‑polymerases have been found as closely related with the ones described earlier [50]. Thus, at first it was suggested that phiKZ-like phages could use bacterial DNA polymerase for their replication. Recently, with the use of modern computational analysis, sequences have been found supposedly coding some proteins, which have a distant similarity with some DNA-polymerases domains, which were found in genomes of several different phiKZ-like phages [51,52].

It is assumed that the mechanism of replication of phiKZ-like phages requires two components, and probably the transition of multiply infected cells into the pseudolysogenic state may be associated with the change of conditions in DNA polymerase activity. For instance, this may be due to deficit of a bacterial component which is necessary for replication. Anyway, the bacterial cell recognizes multiple infection with phiKZ-like phage particles and stops servicing phage replication. This is one of the possible explanations. However, there is another opportunity to develop an adequate explanation for induction in a cell’s pseudolysogenic state after multiple infection with phiKZ and EL-like phages. The genomes of these phages are coded proteins similar to repressors of some temperate phages [50,53]. It is possible that the activity of the gene’s products expressed from a small number of genomes (in low m.o.i.) is not enough to stop or to slow down the lytic development of these phages. An increase in the multiplicity of infection leads to an increase in repressor concentration (more template DNA), which stimulates transfer to a lysogenic condition. In this model, the cell estimates the level of repressor activity and prevents phage development, not with a common mechanism (blocking of a specific site in the phage genome), but with turn-off of bacterial functions necessary for lytic development. In one way or in another, it is evident that in conditions that arise after the infection of cells with high multiplicity, intracellular development of virulent phage is turned off for a while.

**Figure 2:**
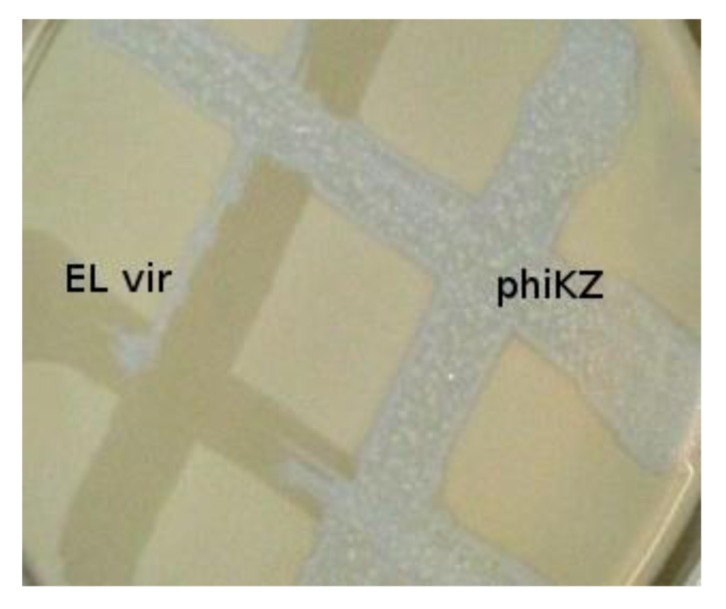
Phylogenetically related bacteriophages phiKZ and EL have repressors of different specificity. Virulent mutant ELvir5 cannot lyse phiKZ wild type infected pseudolysogenic cells (plated on a lawn of bacteria *P. aeruginosa* PAO1).

The phiKZ-like wild type phages are not appropriate components for phage therapeutic mixtures. These phages can lead to HGT (because pseudolysogenic cells can support development of other phages, including temperate ones, transfer of plasmids, and transduction). We have isolated mutants of phiKZ and EL, unable to induce pseudolysogeny and, moreover, actually killing pseudolysogenic cells. The mutants have properties of classical virulent phages. Interestingly, phiKZ and EL behave like phages with repressors of different specificity (with phiKZ revealing dominance) [54], see Figure 2. Probably, the substitution of virulent mutants of phiKZ-like phages for wild type phages in therapeutic mixtures decreases the possibility of HGT [55,56].

**Figure 3:**
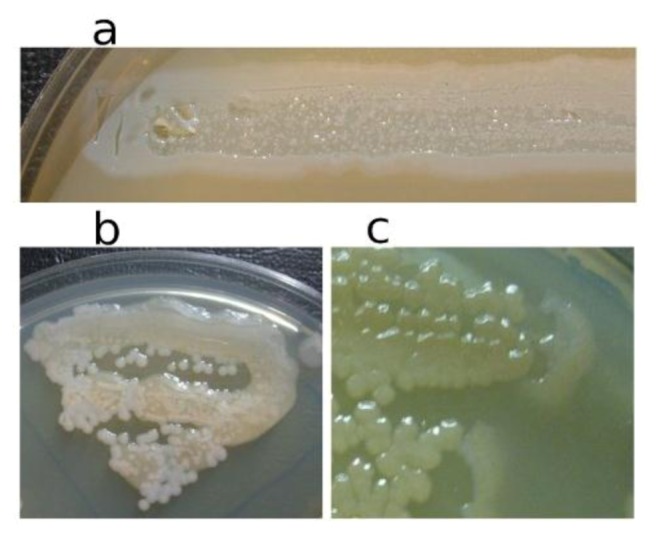
Properties of pseudolysogens which arise after infection of *P. aeruginosa* PAO1 cells with phage phiKZ. (**a**) the growth of phage phiKZ after two days of incubation, the appearance of opalescence in growth of pseudolysogens; (**b**) replanting of the material taken from growth seen on “a” on the surface of the cultural solid medium; single colonies formed with pseudolysogens after two days of incubation. The colonies produced a lot of mucous material; (**c**) segregation of phage sensitive bacteria and their “runaway” of pseudolysogenic colonies.

In Figure 3, the pseudolysogenic growth of *P. aeruginosa* PAO1 cells, infected by phiKZ with high m.o.i. in a Petri dish with nutrient media is shown. Besides the opalescent “bluish” growth of colonies with cells in pseudolysogenic conditions, there are some other additional features, which are of interest. First, material taken from the “bluish” (opalescent) initial growth (Figure 3a), after replanting, produces highly viscous colonies (Figure 3b). One of the possible explanations for such unexpected results may be the conversion of infected bacteria into an alginate producing state. Indeed, as it has been shown, the genes encoding the alginate biosynthetic enzymes are clustered in a single operon, which is under transcriptional control [57]. It may be very important for the activator of the alginate operon is AlgZ, a proposed ribbon-helix-helix DNA binding protein, which reveals some similarity with the repressors of phiKZ and phiEL phages (ORF 196 for phiKZ and ORF 163 for phiEL). Thus, overproduction of phage repressor proteins can be a reason for the activation of AlgZ operon with corresponding alginate overproduction. The second interesting conclusion that can be made from Figure 3c is that after additional prolongation of incubation for several days around the initial growth of opalescent colonies, a growth of phage sensitive colonies with a bluish border can be seen. This means that the sensitive bacteria and phage are able to move a certain distance from the place of initial planting. Phage particles are incapable of active movement. We suggest that phage particles were transported by bacteria resistant (at least for a time) to phage killing ability. One possible reason may be the blocking of intracellular phage development or interference with phage DNA injection (transfer of phages particles on the cell, as a “rider”). The most sensitive bacteria are killed in the process of moving to the border of the colony, as evidenced by a high concentration of phages in the intermediate area.

Pseudolysogenic condition for cells infected with phiKZ-like phages at high m.o.i. in natural conditions may have a biological sense of increasing final phage production. However, the use of these phages for phage therapy in infected wounds may increase viscosity by release of large amounts of DNA. This may obstruct the penetration of other phages or of antibiotics.

### 5.2. Pseudovirulence

The properties of infected bacteria have the ability to influence the development of temperate phages. In some cases temperate phages of P. aeruginosa cannot accomplish lysogenization. For instance Les-phenotype *P. aeruginosa* cells (mutations in control of the recombination genes) cannot be lysogenized with phage D3 [58]. This is a general effect and it can be found in the temperate phages of other bacterial species. For example, the temperate phage GIL01 *Bacillus thuringiensis* has no ordinary C1 repressor to maintain a lysogenic state. Presumably the LexA protein of host bacteria binds to specific 14-bp palindromic sequences within the promoter region of the phage. This prevents phage genes expression in lysogenic bacteria and provides the switch necessary to enter lytic development. Thus any damage of LexA protein creates the condition for lytic development of the phage [59]. 

#### 5.2.1. Pseudovirulence of Phages with Mosaic Genomes and Migration of Non-Transposable Temperate Phage

In the course of classification of phages growing on *P. aeruginosa* PAO1 based on the evaluation levels of genome homology, all temperate phages were assigned to two groups [60]. One group included four species of transposable phages and the other one six species of non-transposable phages. The genomes of temperate non-transposable phages of different species showed a low level of DNA homology in pairwise comparison. The level of homology varied when comparing different phages from different species. Sequencing the genomes of several phages of this group confirmed the presence of homology between them [61,62]. A mosaic structure of genomes was found in general, fairly common in phages of different species of bacteria and usually found in phages active in related species of bacteria [63]. However, there are some examples wherein genomic mosaicism arises through the exchange of genes or blocks of genes between phage of unrelated bacterial species. This suggests the possibility of migration of phage genomes or their fragments between distant species of bacteria; although this transfer could happen before full speciation of the hosts.

We consider several such cases to support this point as well as to look from a different point of view. In study [64], evidence was presented of significant relation at the level of genomes of two temperate phages active on unrelated bacteria, namely *P. aeruginosa* phage phi CTX and *E. coli* phage P2. There are not a large number of genome fragments with complete homology in phiCTX and P2 genomes but pronounced similarity at 28.9%–65.8% is observed in some ORFs. As shown by comparison of the total structure of the genomes of these phages, a high level of similarity is also exhibited. Besides, very importantly, phi CTX and P2 have some similar phenotypic traits, including the similar capsid structure, the lack of response to the effect of inducing agents, and the important feature to accomplish interspecies migration, which is the use of similar lipopolysaccharides for adsorption. Authors of the study suggested that in this case the introduction of intestinal phage P2 in pseudomonade has occurred and considered the case as a clear proof for the ability of phages to overcome interspecific and intergeneric barriers in bacterial hosts. The phage phiCTX is one of the few phage species that is related to the function CRISPR mechanism in *P. aeruginosa* (see Chapter “Temperate *P. aeruginosa* phages and CRISPR effects”). Evidence supporting interspecies migration appears as a result of the isolation and study of new bacteriophages. The phages of *P. aeruginosa* described in [65] are the first representatives of a novel kind of *P. aeruginosa* phages having a similarity in genome structure with N4-like viruses active in *E. coli*. The finding supports the notion of interspecies migrations of bacteriophages.

**Figure 4:**
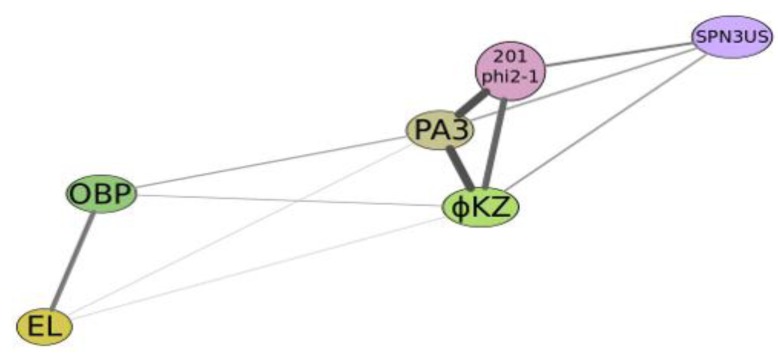
Graphical qualitative layout of the comparative degree of relatedness among a group of phages which reveal some similarity with phiKZ, active on *Psedomonas aeruginosa* (phiKZ and EL), *Pseudomonas putida* (phage Lu11), *Pseudomonas fluorescens* (phage OBP), *Salmonella enterica* (phage SPN3US), *Pseudomonas chlororaphis* (phage 201φ2-1) and *Escherichia coli* (contigs PA3). The thickness and length of connection lines characterize levels of genome relatedness.

Another example of a mosaic genome that arose due to exchange with fragments of the genomes of phages, which belong to different species, including phages active on an unrelated bacterial species, is the genome of phage phi297 (NCBI access number NC_016762) [66]. A significant part of the genome phi297 exhibits a high level of homology to the genomes of phages F116 and D3 of *P. aeruginosa*. At the same time, some special properties F116 and D3 are only partially manifested in phi297. The bacteriophage D3 performs lysogenc conversion of PAO1 surface antigens (serotype O5 in serotype O16) [67]. Such conversion requires the activity of three genes, located in a single fragment of the genome [68]. These ORFs encode three proteins: alpha-polymerase inhibitor; O-beta-polymerase and O-acetylase. Their sequential action leads to a change in the surface structure of lysogenic bacteria and in adsorption specificity. As it turned out, phi297 contains only gene O-beta-polymerase from this group of genes [66]. Apparently, this is not sufficient to ensure complete conversion, and as a result, the phage D3 can be adsorbed onto the surface of the cells of *P. aeruginosa* (phi297) and produce small plaques on its lawn. Further, the two regions homologous to F116, with the coordinates of 1–544 bp, and 47,119–47,438 bp in the genome phi297 match phage integrase gene F116 and its fragments [62]. At the same time, F116 prophage is not integrated into the bacterial chromosome and is present in the cell as a plasmid [69]. Usually, interaction between homologues of the bacterial partitioning proteins ParA and ParB, coded with a plasmid of low copy number, is necessary for stable maintenance and distribution of copies of plasmids in the division of bacterial cells. Phage phi297 genome contains a homologue of parA gene. However, parB is not found in the phi297 genome. Perhaps, lack of parB is the cause of an unstable lysogenization by phi297. Having said that, there is a view that the ParB function is not required for proper distribution of plasmids to daughter cells [70]. Usually, in the newly isolated phages, after sequencing the genome, it is commonly found that plenty of genes coding gene products have no similarities with functions of other gene products in databases. In genome of phi297 there were just eight of such genes. However, even in those cases where phi297 gene products show similarity with some of the gene products available in databases, their real functions are unknown. This is the common case. It can be assumed that in such cases, to determine a value of a gene product for phage viability, it would be useful to apply, along with other approaches, direct genetic research; for example, the selection of mutants of phage with subsequent allocation of the mutations to ORFs. The confirmation of phage genes flowing between these bacterial species has been found in the comparison of phiKZ-like bacteriophages active on different pseudomonades and in the recently discovered large bacteriophage SPN3US active on *Salmonella* [71]. Perhaps this indicates the presence of open-channel exchange between *Salmonella* and *Pseudomona*s. In the structure of phi297 genome there is also definite confirmation of the possibility of migration of large blocks of genes between *Salmonella* and *Pseudomonas*. It would be important to find out the mechanism and ways of such exchanges (participation of unique bacteria, specific bacteriophages, plasmids, migration of native phage genomes, transduction, *etc*.) (see Figure 4).

#### 5.2.2. The Behavior of Wild-Type Phage phi297 on Different *P. aeruginosa* Pathogenic Strains of Different Origin

**Figure 5:**
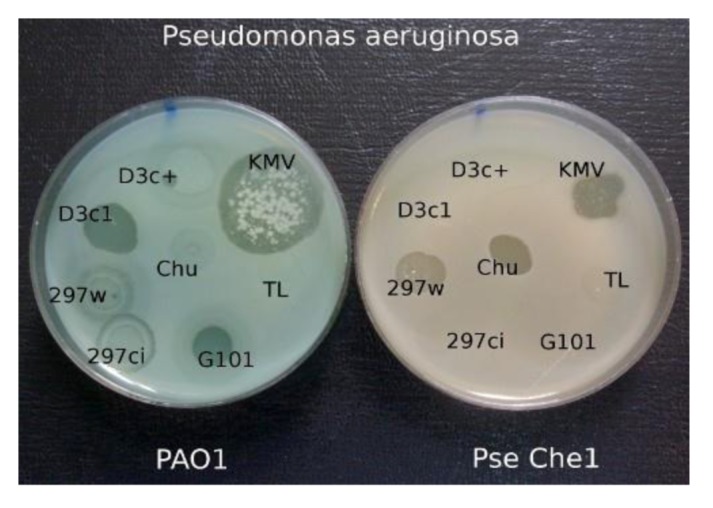
The growth of different phages on *P. aeruginosa *PAO1 and on clinical isolate *P. aeruginosa* Che1. Temperate phage phi 297w does not lysogenize cells of Che1 and behaves as a virulent phage.

When comparing the growth of the various phages on a group of clinical isolates from a burn center, it was found that wild-type phage phi297 grows on the lawns of some clinical isolates in a manner of a virulent phage (see Figure 5). One such strain, Che1, shows resistance to most phages used for the treatment of burn wound infections. Properties of *P. aeruginosa* Che1 have similar properties to Les mutants of *P. aeruginosa* PAO1 (see above), as described in phage D3. However in the case when we accept that *P. aeruginosa* Che1 is indeed a variant of the Les-phenotype, it is necessary to recognize that its Les phenotype is repressor dependant because mutants of phi297 (as phi297ci, phi297vir) with diminished or absent lysogenization capability do not grow on this strain at all. The very existence of these bacterial isolates suggests that such generally accepted concepts, such as temperance and virulence of phages, may lose their ultimate significance in medical practice. Indeed, in the selection of phages needed to treat real infected wounds, it is better to abandon the dogmas that the use, in treatment, of temperate phages is impossible or undesirable because of the danger of HGT. If a temperate phage acts as a virulent in relation to a particular wound pathogen it is obvious that the probability of HGT in such a case will not be higher than for inherent genetic virulent phages.

## 6. Growth of Phages in the Presence of Plasmids

The other examples of disturbances in canonical phage growth types of bacteriophages may be related to extremely diverse and specific effects of different plasmids in clinical isolates of *P.* *aeruginosa.* Conjugative plasmids can migrate between different strains of the same species and also between strains of unrelated species. The plasmids are active participants in the modification of pathogenic islands of bacterial genome transfer gene cassettes, controlling multiple antibiotic resistance, phage resistance or growth modification and genes influencing virulence and pathogenicity. We discuss here some plasmid-phage interactions and their significance for the choice of phages for therapy. The most differentiated activity in phage development inhibition is demonstrated by plasmids of IncP2 group of incompatibility. These plasmids, being specific to pseudomonades, are capable of recombination with IncP1 plasmids of a wide host range and thus can easily migrate to other species of gram-negative bacteria [72]. Such interplasmid recombination provides a powerful means for genetic diversity in plasmids. All IncP2-group plasmids inhibit phage growth, blocking intracellular phage development [73,74,75]. With the use of interspecies crosses between *P. aeruginosa* cells with IncP2 plasmids and *P. putida* it was possible to select plasmid variants with different inhibitive activity. As an example, plasmid pMG53 produced variants with at least six different types of phage growth inhibition. The other IncP2 plasmid, RpL11 also revealed high specificity in phage growth inhibition. It selectively reduces the frequency of lysogenization for temperate transducing phage G101 and transposable phages В39 and D3112 but not for transposable phage B3. It was found that phage D3112 functions, controlling establishment of lysogeny and the lytic cycle, were not expressed after infection of cells with RPL11. However, in the case when the cell carries both plasmid RPL11 and D3112 as prophage, there is no interference with repressor synthesis or with vegetative phage development after prophage induction. Thus, plasmid activity differentiates between the processes of primary integration after infection and that of reintegration of DNA after prophage induction [76]. Moreover, plasmids may recognize fine phenotypic differences among closely related phages with identical repressor immunity. In the presence in cells of plasmid Rms163 (IncP5), transposable phage B39 increase its lysogenization efficiency and as a result has efficiency of plating (e.o.p.) near 0.1, while mutants in c1 gene plate as clear plaques have e.o.p. of 1.0. Two other transposable phages with immB39 reveal a much more profound reaction on Rms163 and practically all infected cells become lysogenized. Mutations in their c1 genes of both phages restore lytic growth (e.o.p. 1.0). The site responsible for such differences in plasmid reaction for immB39 type repressor is located within the interval 1.1–3.9 kb of genome, being closely linked to gene cI [77]. Integration of *P. aeruginosa *transposable phages into plasmid RP4 induces different stable mutations [78,79]. Such hybrid plasmids can migrate among different gram‑negative bacterial species, such as *Escherichia coli*, *Pseudomonas putida*, *Alcaligenes eutrophus* [80,81], they can be recipients for transposable phages of others species, such as Mu [82,83], and can influence the development of phages in other bacterial species [84]. Transfer of plasmid and phage genes capable of causing complicated genome rearrangements and dissemination among the strains as in the environment or as in the microbial wound community, may frequently be the cause of unpredictable results (production of filaments by *E. coli *(RP4::D3112) at 30 °C, stability and growth of *E. coli* (D3112) with a single phage genome copy in chromosome) [85,86,87].

**Figure 6:**
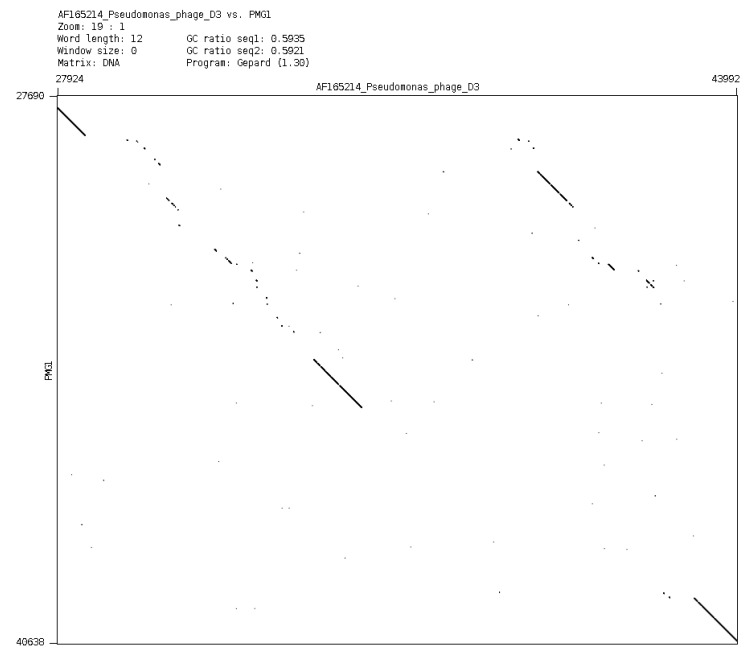
Comparison of phage genomes phiPMG1 and D3 (with Dot-plot program GEPARD) shows the complicated rearrangements in phiPMG1 genome regulatory region. phiPMG1 produces repressor but there are no stable lysogens.

In a general sense, it is possible to consider plasmids and phages as a common gene pool, organized in different structures, designed by nature for the fine regulation of evolutionary events. It is evident that plasmid effects can restrict the possibility of using some phages for the purposes of therapy. However, it is possible to obtain variants of bacteriophages of particular species (mutants or natural isolates), which overcome the inhibitory effect of plasmids. One such example is an isolation of a new phage phiPMG1 (NCBI access number NC_016765), capable to grow in the presence of pMG1, IncP2 plasmid. However, as it happens, phage phiPMG1 can also lyse bacteria with other IncP2 plasmids. It was found that the phage has some other unusual features. Being closely related with temperate phage D3, it cannot make lysogenic bacteria. Results of phiPMG1 genome sequencing [88] have shown (see Figure 6) that in the central part of its genome some very complicated restructuring events have occurred. In spite of its repressor gene being undamaged, the phage cannot accomplish stable lysogenization as D3 does, but only induce a temporary lysogenic state in infected cells.

Such observed virtual virulence of the phage has arisen as a result of gene rearrangement in the regulatory region of the genome. Presence of plasmids can block lytic growth of recognized therapeutic phages. For example, plasmid RMS148 interferes with lytic development of phage phiKZ of *P. aeruginosa*. This fact has permitted the revelation of the transducing activity of phiKZ. It means that in the course of phage therapy that particular virulent giant phage may transduce in its own particle up to 5% of *P. aeruginosa* bacterial chromosome [89]. It is more than the largest of known pathogenic islands in genome of *P. aeruginosa* and more than the sizes of temperate prophages able to participate in construction or modification of pathogenic islands.

## 7. Possibilities for the Emergence of New Phages in the Course of Phage Therapy

It may be interesting to estimate the probability of the emergence of new recombinant phages as a result of the application of phage therapy to wounds infected with pathogens of different distantly related species. For example, *Pseudomonas aeruginosa* and species of *Burkholderia* complex often coexist in places of common infections (wounds, CF). For both bacterial groups similar temperate transducing phages are described. There are temperate phages specific for *Burkholderia sp.* growing on *Pseudomonas aeruginosa* [90] and there are strains of *Burkholderia*, which support the growth of temperate phages *P. aeruginosa *[44]. We see examples of interspecies migration for phages of several different types, including (1) transposable phages *P.aeruginosa*; (2) converting temperate phages related D3; (3) phage F116.

### 7.1. Transposable Phages

In our previous studies, we found plenty of different transposable phages of *P. aeruginosa* (all of them are temperate), which were distributed into groups of D3112-like phages (14 phages, one species) and B3-like phages (six phages, three species) [91,92,93,94]. 

D3112-like and B3-like phage genomes are composed in a similar pattern, but mostly from different non-homologous blocks. Phages belonging to different groups reveal only traces of DNA homology along most parts of their genomes. Some essential DNA homology can be found on the right ends of their genomes where genes responsible for specific adsorption to bacterial IV type pili are located [95]. Nevertheless, it has been possible to isolate several D3112/B3 hybrid phages with very strange genome structures. They have arisen through an even number of crossovers with low frequencies. The comparison of D3112 and B3 genomes by dot-plots has found just several of the 30–50 bp long DNA homology sites distributed along the genomes. We suggest that these small sites of homology are used in interspecies recombination. The compulsory even number of crossovers may be explained by the possible incompatibility of specific gene modules in D3112 and B3 [96,97]. D3112-like and B3-like phages have a difference in their transposition specificity. Although phage D3112 has multiplicity of the integration sites in *P. aeruginosa* chromosome and plasmids [79], nevertheless, it does not induce auxotrophic mutations in lysogenized bacterial cells. However, phage B3 and its relative PM105 induce such mutations with different nutritional requirements [98]. We have suggested that D3112 has a high transposition specificity and mostly uses sites that are located in the part of the *P. aeruginosa* chromosome controlling catabolism. Indeed, Rehmat and Shapiro [99] have localized different D3112-induced mutations in the amidase gene.

It was found that genome of D3112 may be expressed in different species of gram-negative bacterial species (*Escherichia coli*, *Pseudomonas putida*, *Alcaligenes eutrophus*) when delivered as a part of hybrid plasmid RP4::D3112 [80,81,85,86]. In the new hosts, the expression of D3112 can change some of the bacterial properties. Thus, the hybrid strain *E. coli* (RP4::D3112) grows well only at 42 °C but dies quickly at 30 °C (forming long filaments as a result of blockade of cell division). 

The real reason is that D3112 in *E. coli* cannot establish stable lysogenic conditions but its transposase is active. However, at 42 °C D3112 genome transposition is blocked, and cells survive and form colonies [86]. It is possible to isolate quite stable *E. coli *strains where phage genome is inserted into bacterial chromosome with loss of RP4 plasmid. Such *E. coli* (D3112) clones produce a very low number of mature D3112 phage particles. It means that in such clones, a foreign phage becomes a component of *E. coli* genome and its stability is ensured by rare expression in single cells. It may be considered one of the examples of phage migrations. Such phage interspecies migrations (including migrations of plasmids with complete phage genomes or their fragments) may lead to emergence of new pathogens. 

There are some other effects related to activities of transposable phages of *P. aeruginosa*. It has been shown that after thermal induction of *P. aeruginosa* cells carrying heat inducible prophage D3112 cts15, it is possible to observe plenty of different survivors producing highly mucous colonies [100]. Most of them quickly (after 1–2 replatings) lost their mucous phenotype but some of the survivors showed good stability. 

Frequently among related transposable phages the effects of mutual influence on vegetative growth may be found. This is usually found in the interaction of phages of the same species, but with different specificities of repressors; for example, the suppression of the vegetative growth of phages with immB39 on the lawns of bacteria carrying prophage with immD3112. The suppression was found to be determined by the activity of locus *cip. *The *cip* is located in the genome of D3112 in the range of 1.3–2.45 kb and reveals its inhibitive effect only in lysogenic bacteria. In the case of multiple lysogeny the suppression increases substantially. There have been isolated mutants of phage B39 which can not be inhibited with Cip-activity. Mutations in gene cip which lost the ability to suppress B39 also can be selected [101,102].

As was found in the study of the genomes in different *P. aeruginosa* transposable phages, all of them have a pronounced modular structure that shows evidence of the possibility of free exchange with gene modules between the genomes of phages of the same species. Interestingly, in some individual genes, their composite nature can be detected. So, the number of phage repressor genes is composed of three sub-modules, which are supposed to control the various functions of repressors [103,104]. Apparently, the genomes of *P. aeruginosa* transposable phages, even those which now show no homology along their genomes genes (phages of D3122 and B3 groups), have the single pattern of a genome of an ancient predecessor. This is confirmed firstly by a similar sequence in groups of genes controlling similar functions; secondly, by the presence of a considerable number of genes with good homology which control the flexible structure of the tail and its ability to adsorb to bacterial pili. However, transposable phage BcepMu, active on *Burkholderia cenocepacia*, has a different origin, because it reveals some common features with phage Mu of *E. coli *(they have a contractible tail), although they have differences in specificity transposase compared with the phage Mu [105]. Likely divergences in the evolution of different types of phage-transposons for gram‑negative bacteria occurred at the moment of selecting the main host. It is possible to suggest that most of the time the genome evolution of BcepMu like phages was in bacteria belonging to the family Enterobacteriaceae. In the genome BcepMu are present homologs of many genes of different strains of salmonella prophages.

We can assume that the differences in the genomes of modern *P. aeruginosa *transposable phages belonging to different groups arose as a result of their numerous migrations. This is confirmed by the results of genome annotation of transposable phages. Thus, in D3112 phage genome there were found genes which are orthologs and not only genes of pseudomonads, but of other bacteria or phages. For example, ORFs, 18, 19, 24 and 26 of D3112 encode proteins similar to protein defective prophages PNM1 PNM2 and two different strains of *Neisseria meningitidis* MC58 and Z2491, the next group ORFs 27, 28, 29, 32, 34 encodes proteins similar to proteins of the phage Mu, *etc*. [106]. In case of phage B3 it was found that its proteins have mostly another origin, revealing relatedness with proteins of phage Mu, BcepMu or *Salmonella typhimurium*. In accordance with results of phage B3 genome annotation, not less than 10 genes have their origin from genes of other phages of different bacterial species: five genes of salmonella phage P22, five genes of two BsepMu-like phages and one gene is a homologue of the gene in salmonella phage Sti3. Genes found in phage B3 genome reveal different levels of relatedness (on the level of controlled products) with such potential intermediate bacterial hosts as *Bordetella bronchiseptica*, *Burkholderia cenocepacia*, *Escherichia coli*, *Haemophilus ducreyi*, *Haemophilus influenzae*, *Neisseria meningitidis*, *Salmonella enterica*, *Vibrio cholerae*, soil species and plant pathogens of different families including non-pathogenic and pathogenic strains with various ecological niches [107]. In this respect, it should be interesting to continue the study of two other B3‑like species of transposable phages, active on *P. aeruginosa*, but with a significant difference of B3 in the level of DNA homology [93,94]. It may be especially interesting considering the significance of *P. aeruginosa* transposable phages in CRISPR-effects (see later). 

The origin of several phage genes in D3112 and B3 cannot be determined through annotation. To elucidate their functional significance in phage development it is necessary to accomplish detailed phenogenetical studies (directed mutagenesis of such genes, looking for mutants with a specific phenotype and study of their behavior under different conditions).

### 7.2. Converting Temperate Phages of P. aeruginosa

Temperate *P. aeruginosa* phage phiCTX carries a cytotoxin gene which is expressed in prophage state. The phage has a large genome homology with *E. coli *phage P2 as a result of highly probable interspecies migration. Both phages are identical in particle morphology and their genomes have similar structures. However, the fact that genome of phiCTX is similar in GC-content with *P. aeruginosa* suggests that the divergence of P2 and phiCTX occurred a long time ago [64].

**Figure 7:**
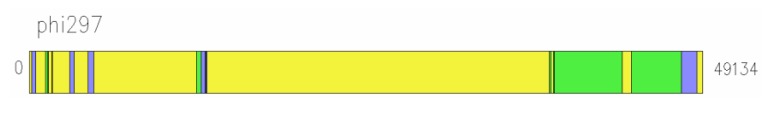
Genome of phage phi297 has homology regions of different sizes and locations with D3 and F116 genomes (yellow: phi297 own genes; green: fragments of homology with D3 DNA; blue: fragments of homology with F116 DNA).

D3 is the other converting temperate phage of *P. aeruginosa*, which in the prophage state changes the structure of the surface lipopolysaccharides, and prevents adsorption of several bacteriophages, including D3 itself [61,68]. Phage phi297, showing DNA homology to DNA of D3 and of F116 [61,108], also has converting activity. The distribution of DNA homology regions in genome of phi297 with genomes D3 and F116 is shown in Figure 7. The mosaic structure in the phi297 genome is evident. Phi297 inherited from phages D3 and F116 just some of the genes that give these phages unique features, including the ability of D3 to perform bacterial surface modification and, possibly, the ability of phage F116 to be plasmid in prophage conditions. As a result, phi297 carries only a partial modification of cell covers (as shown by only partial blockade of phage adsorption). At the same time, as it is proposed, inheriting only one of the F116 genes, necessary to support prophage as plasmid, phi297 phage does not form stable lysogens.

We have found that phi297 is capable of recombination with the bacterial PAO1 strain chromosome, producing lytic non-reverting variants, incapable of accomplishing lysogenisation. The resulting hybrids exhibit lytic properties towards certain groups of clinical isolates and phage resistant mutants arising after use of commercial phage mixtures [66,88]. We consider that the addition of such lytic derivates of temperate phages into commercial phage mixtures for the cure of infected wounds may be useful for enlarging their spectrum of lytic activity. The lytic activity of the hybrid phi297vir will stimulate the search for other similar possibilities. It will require more detailed studies of temperate phages of different species and the isolation of new species of temperate phages.

### 7.3. Phage F116: Interspecies Wanderer?

Bacteriophage F116 is of special interest for the assessment of the possibility of experimental and inter-specific migration and study of its mechanisms. The fact that F116 genome fragments (or an intact genome of other phages with great similarity to the genome of F116) were found in an integrated state in *Neisseria *support the need for special attention on the further study of F116-like phages. Indeed, pathogenic *Neisseria gonorrhoeae* strains do not support growth of any of the presently known tailed phages. However, as a result of careful studies in bacterial genomes, several regions were found showing similarity to genes of different phage species. One of these regions, NgoPhi2 is unique in showing a very high level of similarity with the genome of the phage F116 [109]. Genes of prophage, which reveal relatedness with F116, are apparently intact, and exhibit multiple effects, including the inhibition of the growth of *E. coli* and the propagation of phage lambda. The repressor in the NgoPhi2 region was able to inhibit transcription genes of *N. gonorrhoeae* and of phage *Haemophilus influenzae* HP1, and the gene for choline replaces the function of the homologous gene of phage lambda. After induction of cells with mitomycin C and microscopy of the supernatant, complete phage particles were found. It can be assumed that the phage genome exhibiting affinity with F116 was introduced into *N. gonorrhoeae *by an act of HGT (in the course of direct infection with phage particles, or in the state of prophage plasmid in the conjugation process or further being integrated into a transmissible plasmid).

### 7.4. Evidence for Migrations of phiKZ-Like Phages

Bacteriophage phiKZ active to *P. aeruginosa *reveals such features as large particle size, large genome and a spiral formation, the “inner body”, and a unique packaging of DNA as spools of thread [45,46]. Later two other species were described of giant phages of *P. aeruginosa*, EL and Lin68, which are not distinguishable from phiKZ in particle morphology [47,48]. Recently, structural proteomes of phiKZ and EL were studied in detail [110]. However, all new phages exhibit significant differences from phiKZ. Phage EL has no detectable genome homology with phages of the other two species, and differs from them in GC-composition and in size of the genome [53]. Phages of another species, Lin68, show DNA homology of a low level with phiKZ in one of the restrictional DNA fragments [47]. In addition, phages of species Lin68 are unable to grow at 42 °C, and their mature particle reveals more instability than phiKZ particles at 60 °C. The phages of this species exhibit weak lytic activity on the lawn of psychrophilic bacteria *P. fluorescens*. It is of interest to compare the genomes of phages in the species Lin68 with phiKZ-like phages of other psychrophiles. The phiKZ-like phages can persist in pseudolysogenic clinical isolates in wounds. Thus, phage Che, a new EL-like phage, has been found in a clinical isolate from the Chelyabinsk burn center. *P. aeruginosa*, at least in other bacteria; similar phages were not found for a long time. However, as has been shown, bacteriophages of similar morphotype, showing signs of kinship with phiKZ, can be found in other bacterial hosts [48,51,111,112,113]. It is possible too that phiKZ-like phages will be found among other large phages of distant bacterial species. A recently described giant bacteriophage specific to *Salmonella* [71] and another one for *Ralstonia solanocearum* [114] have similarities with phiKZ-like phages. Apparently, the ability of all phiKZ-like phages to establish a pseudolysogenic state in infected cells has evolved from an ancient temperate phage as a result of successive multiple migrations between different hosts. If it is possible to confirm specific DNA packaging for these phages as inner bodies, as in phiKZ, it will prove not only the existence of a new phage superfamily, but also the existence of the open gene exchange channel between *Pseudomonas*, *Salmonella* and soil types of bacteria for this phage superfamily.

## 8. Optimizing the Selection of Phages for Therapy

The detailed phenogenetical study of a phage is a compulsory condition for its acceptance for direct use in phage therapy or inclusion into phage therapeutic mixtures. Although in each case the approach for this study may be different, the first aim is to show that a selected phage is genuinely virulent. In case the phage virulence is derivative of a temperate phage, it is necessary to prove its incapability to revert into a temperate condition in different situations (as multiplicity of infection, temperature, presence of plasmids or temperate phages, *etc*.). For example, the giant phiKZ-like phages of *P. aeruginosa* are used in various commercial mixtures [108] due to their wide spectrum of lytic activity. These phages in standard single-stage growth cycle experiments behave as typical virulent phages by lysing all infected bacteria, but at a high multiplicity of infection, bacteria continue dividing over several days (see above). During this time, the infected cells can be recipients of HGT through the activity of plasmids or temperate phages, which often occurs in wound microbial communities.

The number of genuinely virulent phage species for *P. aeruginosa* is quite limited. There are only nine species of different families, and, moreover, some species may have limited usefulness. In the list below are the phage species (in parentheses are the minimum and maximum size of genomes of the species, where it is known). Family Podoviridae: (1) the species of phiKMV-like (42,954–43,548 bp); (2) species Luz24-like (45,503–45,625 bp); (3) species N4-like (72,544–74,901 bp); (4) species LUZ7/PEV2*; family Syphoviridae: species (5) M6-like (58,663–59,446 bp); family Myoviridae: (6) species PB-1-like (64,427–66,530 bp); (7) species phiKZ-like (280,334 bp); (8) species Lin68 (is not sequenced); species (9) EL-like (211,215 bp). Different manufactures can use one or the other species. However, it is evident that the number of species is not unlimited.

Of this list phiKZ-like phages (two species) can be considered only as conditionally virulent. Virulent phages of other species—Luz24 and TL—exhibit high relatedness with temperate phage PaP3 [115,116]. So far the most promising species for phage therapy of P. aeruginosa infections are phages of species PB1-like phages, which exhibit significant variability on the basis of the specificity of adsorption [117,118,119], and species of phiKMV-, N4-, LUZ7- and M6-like phages. Bacteriophages of species Lin68 can have only limited application, being sensitive to the development of human body temperatures. We can conclude that the phage potential of *P. aeruginosa* is limited. Variations in lytic activities for phages inside each of the virulent species are also limited. A sequenced set of phages and their number are altogether insufficient to maintain long-term phage therapy. Obviously, the need to expand efforts to find new species of virulent phages exists. Moreover, there must be some coordination of specialists working with phages and interest in the development of phage therapy to overcome the randomness in the selection of phages for sequencing and phenogenetical studies.

For each prospective therapeutic phage it is desirable to estimate the probabilities and diversity of phage-resistant mutants. According to our observations both evaluations vary greatly in dependance on phage species and (obviously) on specific hosts: clinical isolates and laboratory variants of the standard host.

## 9. At First Glance, the Phages with a Broad Spectrum of Lytic Activity Are Preferred for Therapeutic Blends, but only at First Glance

The use of sophisticated phage cocktails, including many strains of phages with a broad spectrum of lytic activity against different bacterial pathogens, has as its purpose the fast interruption of infection without prior isolation of the pathogen. This is justified only in cases where the infection and the loss of time may create a life threatening situation. However, it may be that the long-term presence of pathogenic bacteria in the wound are caused by the unique bacterial strains that are resistant to most of the phages in the therapeutic mix. Because there is no previous information about strains in the wound, before applying emergency treatment with a phage mix, in fact, the attempt at treatment iscarried out blindly and if the first application of phages does not give positive results, it means that time is lost. However, in the majority of cases of wound or nosocomial infections due to *P. aeruginosa*, there is usually no such extreme urgency.

Typically, the clinical laboratory identifies pathogenic bacteria bv determining their sensitivity to various antibiotics and only if antibiotic application is ineffective, will the decision be made on the use of bacteriophages. Thus in the case of wound and nosocomial infections in different locations due to isolated strains, more rational is the use of a minimal set of phages, even a single phage with a narrow spectrum of lytic activity capable to lyse strains isolated from the patient. The choice of a set of phages or a single phage against a particular pathogen is not a more labor-consuming procedure than a standard check of bacterial sensitivity to antibiotics with paper discs. Such a restriction in the number of phage species is intended to limit the accumulation of mutant pathogens that are resistant to the most valuable phages with a broad spectrum of lytic activity and save them for use in really extreme cases. Thus bacteriophages with a narrow spectrum of lytic activity can also be very useful in phage therapy. We consider the properties of several of these phages.

## 10. Description of the Properties of New Phage in the Possible Application of Phage Therapy

### 10.1. The Properties of a New Bacteriophage CHU Pseudomonas aeruginosa

**Figure 8:**
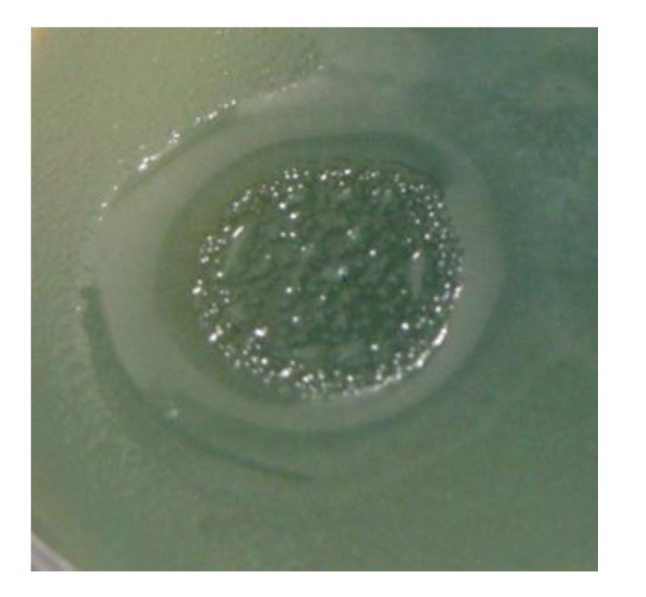
The raised slimy ring on the CF013A lawn around the phage CHU lysis spot.

Phage CHU is one of the newly discovered phages for *P. aeruginosa*. This is a relatively small (diameter of the head is near 50 nm) DNA containing tailed Syphoviridae phage. The unique feature of the phage is an unusual spectrum of lytic activity (trace growth on PAO1 strain and good growth on several strains producing alginate). There are two such strains, Pse163 and CF013A, isolated from CF patients (from the collection of Prof. M. Vaneechoutte, Laboratory Bacteriology & Virology University Hospital Ghent, 9000, Gent, Belgium). These strains differ in their phage sensitivity and other properties (Pse163 is lysogenic for a transposable phage related with D3112). Phage CHU grows on lawns of washed alginate Pse163. The phage has no alginase activity, as there is no visible destruction of slime around the spots of phage growth. There is, however, a difference in the character of phage CHU growth on lawn of strain CF013A. After a prolonged (three days) incubation around a lysis spot CHU on the lawn of CF013A was formed, (see Figure 8). The reason for this effect may be related to the difference in properties of alginate producing strains. It was confirmed in the study of four additional stable P. aeruginosa alginate producers exhibiting selective sensitivity to phage CHU, which were found among clinical isolates of the burn center of the Chelyabinsk Regional Hospital. All these four strains grow similarly, being plated on the surface of the agar medium. However, when plated in semisolid agar layers, two of these strains do not produce a visible layer of alginate but the other two strains continue to produce alginate, visible on the surface of the upper agar layer. Bacterial strains, which do not produce visible alginate, on being placed into semisolid agar of the upper layer, reveal alginate around phage CHU spots of lysis. However, the reasons for the difference in properties from two kinds of alginate producers, remains unknown. As the last four strains from the burn center were isolated at different times from different patients they may be regarded as hospital pathogens. It is possible the differences between them arose in the burn center.

**Figure 9:**
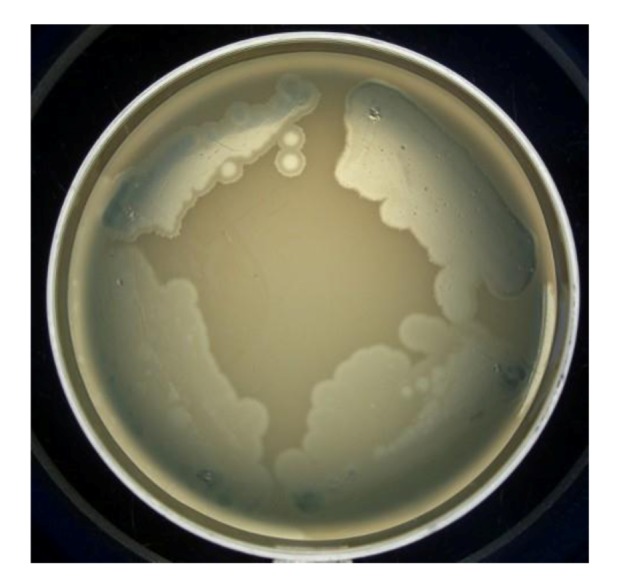
Growth of CHU phage variants on the lawn *P. aeruginosa* 8–20.

A feature of phage CHU is the high level of instability. The best differentiation of plaque types was achieved with use as lawns of strains *P. aeruginosa* 8–20 and 2–10 (obtained from Professor C. Pourcel, GPMS Institut de Genetique et Microbiologie Bat 400 Universite Paris-Sud 91405 Orsay cedex, France), see Figure 9. Up to now, despite repeated attempts, we could not isolate stable plaque derivatives of phage. In this relation CHU reveals some similarity with phage TL, described below. 

The use of this phage can be useful in open infected wounds against strains producing alginate.

### 10.2. Properties of a New Bacteriophage TL

**Figure 10:**
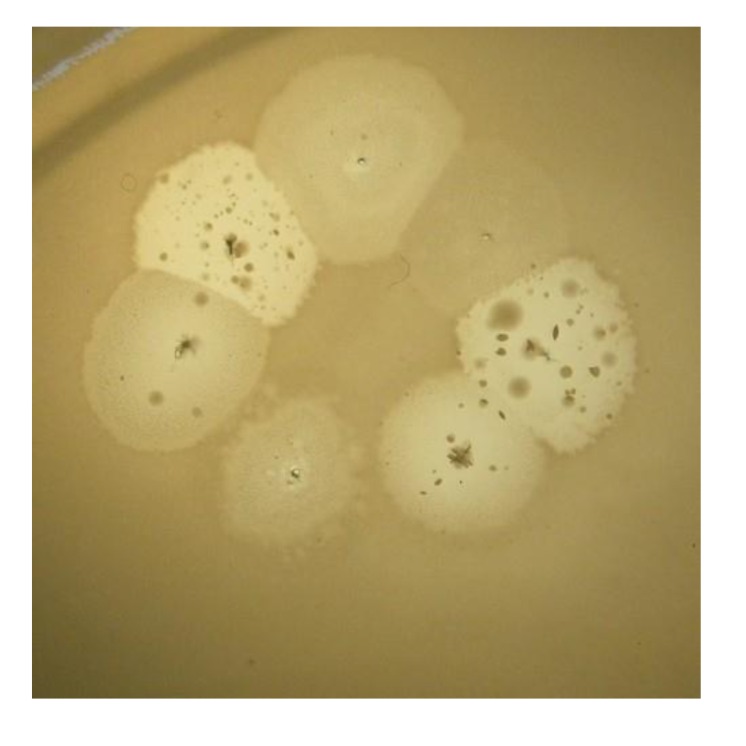
Growth of TL phage variants on the lawn *P. aeruginosa* ELR2.

Bacteriophage TL genome sequence (carried out in the laboratory of Professor L. Kulakov, (The School of Biological Sciences, The Queen’s University of Belfast, Belfast BT9 7BL, Northern Ireland, United Kingdom), annotation made in our laboratory shows a high degree of relationship, with phage Luz24 [115,116]. TL reveals a relationship (as Luz 24) with the temperate phage PaP3. This leaves no doubt as to the common origin of the three phages. TL has poor growth on lawns of strain PAO1. A PAO1 mutant ELR2 has been isolated which is resistant to many phages, but supports good growth of TL. We suggest the loss or modification of surface adsorptional receptors led to the availability of receptor for phage TL [35,117,118,119]. Phage TL is produced on the lawn of ELR2 plaques with a different appearance, which cannot be stabilized, see Figure 10.

**Figure 11:**
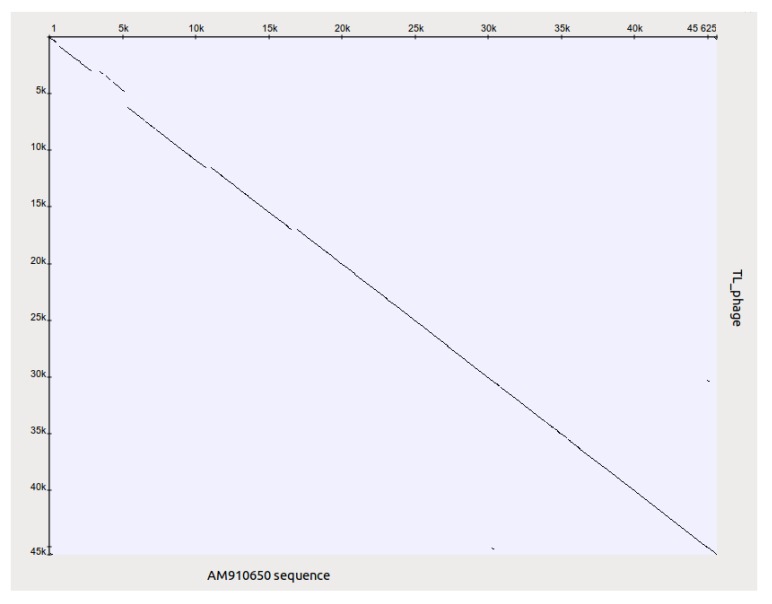
Genomes of phages TL and Luz24 have a high level of DNA homology (DotPlot on UGENE program parameters: 60 bp, 85% similarity) but TL genome contains transposase gene in the region of non-homology with Luz24.

After the genome annotation, it was found that the TL genome contains gene coding for transposase gene in the region 5,000–6,000 bp. The gene was not detected in genomes of related phages Luz24 (AM910650), see Figure 11, and PaP3. The appearance of different plaque morphology variants and their instability is may be due to the activity of the phage transposase. As TL does not produce lysogens, like Luz24, it can be attributed to the lytic phages. TL is capable of lysing bacteria resistant to other phages used in anti-*P. aeruginosa* preparations. Therefore, the introduction of TL into any standard phage mixture against *P. aeruginosa* may be very useful. Indeed the phage will reveal its activity when the wound *P. aeruginosa *pathogens become resistant to other phages in the mixture. Although the features of this phage confirm the importance of the proper selection of phages in the preparation of the phage therapeutic mixture, nevertheless the use of Luz 24 related phages in phage therapy requires a more thorough study.

## 11. Significance of Specific Phage-Bacteria Interactions in Transfer to Personalized Phage Therapy

The mixed microbial community includes different types of bacteria (pathogenic and nonpathogenic) which may be in complicated mutual relations. For example, in the case of cystic fibrosis the frequent companion to *P. aeruginosa* is the bacterial species of the *Burkholderia *complex. One of the reasons for the persistence of *Burkholderia *is the presence of alginate [120]. There are described cases of acquisition, of the ability to grow at 37 °C, adhesiveness and cytotoxicity comparable with the corresponding features of *P. aeruginosa,* by psychrophilic soil pseudomonades as *P. fluorecens*, which were previously considered as harmless [121,122]. Furthermore, strains of *P. putida* with cassettes controlling antibiotic resistance in plasmid-borne integron from an *Achromobacter xylosoxidans *were isolated from several patients in the general intensive care unit, which suggests the possibility of evolutionary interactions between commensals in the clinical conditions [123]. It can be expected that the frequency of finding such new species from commensals will expand as will their significance in wound bacterial communities (carrying with them a set of new genetic elements—phages, plasmids, transposons—that can perform HGT). Thus, there is a need to continuously increase the number and diversity of bacteriophages that are acceptable for the treatment of infected wounds.

Accordingly, the number of truly virulent phage species is usually small in the case of different bacterial species, including *P. aeruginosa*, which obviously limits the possibilities of phage therapy. Moreover, it is clear that the continued use of the same phage will quickly lead to the emergence of multiple phage-resistant bacterial pathogens. It is impossible to predict the time of active use of the described phage species in the case of widespread introduction of phage therapy. It is not evident that we must expect to reveal a sufficient number of new virulent phage species. In this regard, there is a need to understand and accept decisions on the possibility of using specially modified temperate phages in phage therapy.

## 12. Let Us Discuss the Possibility to Use *P. aeruginosa* Temperate Phages in Phage Therapy

Temperate phages active on *P. aeruginosa* make up a significant part of the phage potential of this species. Refusal to use temperate phage of wild types capable to lysogenize infected bacteria is understandable and evident. However, as we have shown, among the recognized phage species acceptable for therapy, there are phiKZ-like species whose behavior (lytic against pseudolysogenic) may greatly depend on cotrolled or uncontrolled conditions in their application. In the case of these phages, special virulent mutants incapable of pseudolysogenization have been isolated.

We consider that it is possible in certain cases to use some temperate phages after their inheritable modifications or even to use phages showing temperance in infection of standard laboratory hosts, but having obvious virulence for real wound strain. Of course this can be done in conditions of individual selection of specific phage only under the supervision of physicians and clinical laboratories. Quite a lot of temperate *P. aeruginosa* phages have sequenced genomes. The most studied phages belong to the Syphoviridae family, including species: (1) transducing cytotoxic phiCTX-like: phiCTX (35,580–36,415 bp); (2) D3-related: D3 (56,425 bp), PAJU2 (46,872 bp), phi297 (NCBI HQ_711984), phiPMG1 (NCBI NC_016765); (3) F116-(65,195 bp); (4) Transposable phages: (a) D3112-like (13 different isolates): D3112 (37,611 bp), MP22 (36,409 bp); DMS3; (b) B3-like phages (six phages) B3 (38,439 bp), HW12, PM105 [93]. In addition, there is a group of phages of different families and species, which should be studied in more detail as Myoviridae, P2-like-MP29, MP38, F10; Podoviridae 119X, PaP2; Syphoviridae: 73, G101, 160 and unclassified phage PA11.

Phage phiPMG1 may be an example of an earlier temperate (D3-like) phage, which became acceptable (in our opinion) for use in therapy as a result of spontaneous genetical modifications [88]. Our attempts to isolate revertants of the phage similar to D3-like wild-type phage were unsuccessful. We tested the activity of this phage compared to the activity of two commercial mixtures of different manufacturers and showed that this phage successfully lyses spontaneous mutants of strain PAO1, resistant to phages in both commercial mixtures [108]. Phage phi297vir, which is a recombinant of phi297 wild type with bacterial chromosome also lyses resistant to commercial mixtures of bacteria, including bacteria in the pseudolysogenic state after multiple infections with phiKZ-like phages, such as bacteriophages.

The study of new species of temperate phages expands our understanding of their evolution [64]. For instance, the mosaic structure of phi297 genome occurred as a result of directly borrowing gene encoding products that are similar to the products of bacteria and phages of different taxonomic groups (including *Salmonella* and other enteric bacteria). This clearly supports the idea that genomes of different natural phages have arisen as a result of a combination of pre-engineered functionally related genes. Introduction to the temperate phage genome modifications, leading to irreversible virulence, can be used to expand the collection of therapeutic phages.

Of course, the likelihood of HGT using even non revertible virulent variants of temperate phages will be higher than in the case of truly virulent phages (because, for example, of the possibility of such a mutant recombining in infected wounds with related prophage in pathogenic bacteria). However, the immediate danger to the patient in the course of phage therapy will not be raised because the duration of phage therapy is usually not more than a few days. If during this time, the therapeutic effect is not achieved, it means that the mixture of phages in this particular case is not efficient and should be replaced [37]. Phage phi297vir behaves as lytical but it is not virulent, since it does not grow on bacteria which contain wild type prophage phi297w. Apparently phi297vir remains a functionally active operator.

## 13. Temperate *P. aeruginosa* Phages and CRISPR Effects

We have discussed the above possible migrations of intact phage genomes or their fragments between bacteria of different species. One of the obvious ways is the migration of phage genomes incorporated in the conjugative plasmids, as shown for transposable phages of *P. aeruginosa* (see above) and *E. coli* [123]. Another possible means for migration may be acceptable for phages by using for their adsorption type IV pili. Type IV pili are highly specialized structures on bacterial surfaces having immense significance in bacterial life in the expression of many phenotypes, such as motility, sensitivity to bacteriophages, natural genetic transformation, adherence and speciation for multicellular behavior. Modified bacterial pili of IV type may provide adaptation to specific tissues expanding the possibilities of the particular species of pathogenic bacteria and increasing their virulence [124,125,126]. The pili, being frequent appendages of different pathogens and adsorptional receptors for numerous phages (as F116, transposable phages of *P. aeruginosa*, possibly transposable phage specific for *Neisseria meningitidis*), may be used by such phages in interspecies migrations [127,128]. It is possible to assume that this interspecies migration path made the bacterial cells develop CRISPR as a specific mechanism of protection against expression of foreign genetic material, plasmids and phages. Loci CRISPR, control the synthesis of specific small RNA molecules that, under invasion by viruses or other genetic elements block their expression, binding the foreign DNA. The number and variety of such phage spicers evidences the number of previous contacts of bacteria with different foreign DNAs. At the same time, there is evidence that the CRISPR mechanism, a multifunctional system response, manifests itself not through the blockade of phage or plasmid penetration to save individual cells subjected to attack, but by modifying other essential features. Infection bacteria *P. aeruginosa* PA14 with phage DMS3 inhibits biofilm formation and swarming motility, both very important bacterial features associated with the activity of type IV pili. After introduction, mutations in the CRISPR region of both biofilm formation and swarming in DMS3 lysogenized strains recovered [129].

Reports of the suppression of the growth of temperate phages are rather contradictory. Some studies clearly stated the suppression of the growth group of temperate phages, which (according to the results of the comparison with database) are mainly related to the species transposable D3112-or B3‑like phages and some other species as F116 and phiCTX. In other works, the inhibition of the growth of phages is denied. Thus, in two moderate phages, MP29 and MP42, whose genomes are similar to those of *P. aeruginosa* transposable temperate phages DMS3, there was no change of swarming [130]. Such discrepancy in results from different research groups may reflect subtle differences in the structure of phage genomes, as it is believed even point mutation in the genome of the phage can eliminate the effect of its CRISPR-suppression mechanism [131,132]. Strains in different laboratories are used under one name, which derived from the same parental strain, can rapidly accumulate point mutations, with unpredictable effects. Therefore, the hopes of using such a labile system for practical purposes (e.g., in the case of cystic fibrosis) may be considered premature. There is evidence that *P. aeruginosa *PA14, being capable of preventing replication of six newly isolated temperate phages can also acquire a new spacer content as a result of some lytic phage infection. The authors consider it as evidence of the adaptive nature of this CRISPR/Cas system [133]. CRISPR‑bearing loci of the strains of *P. aeruginosa* are quite common among clinical isolates [134]. For example, among 122 clinical isolates sampled in different geographical areas 45 strains were found carrying CRISPR with 132 viral spacers matched to temperate bacteriophages/prophages capable of inserting into the host chromosome, but not to extrachromosomally replicating lytic *P. aeruginosa* bacteriophages. It is unclear exactly what the authors define as lytic phage. Because none of the tested temperate phages showed deterioration in growth or lysogenization in the presence of CRISPR, described in this paper, the authors actually reject the dogma for CRISPR protective effect.

The examination of the spacers (inserts) in CRISPR loci, found in all strains of *P. aeruginosa* studied to date that this system is aware of, only several definite temperate phages. At least for most of the phages, bacterial pili are compulsory receptors of adsorption. These pili are very important for the establishment of relationships in the microbial community. Bacterial conjugation is a form of such relations, carried by IV type pili in which DNA and proteins are transferred to the appropriate recipient cells [135].

A common feature of these phages is their high mobility. An existing relationship between the bacteria and most temperate phages are relations in a community where all interactions maintain a common gene pool and adapt to changing environmental conditions through natural selection (including humankind with its various activities as an integral part of the environment). There is an opinion that CRISPR activity is not designed to preserve the functional capabilities and integrity of the bacterial genome in a single bacterial cell. Moreover, it is protection from the expansion of destructive effects on all cells in a population, possibly in a state of biofilm. On the other hand, CRISPR can be viewed as a mechanism of active accumulation in the bacterial genomes of different phage genome fragments of different phages. It may create the possibility of re‑infecting bacteriophages for further evolution through recombination. This may be of general interest, as there are many clinical isolates supporting the lytic cycle of temperate phages but not lysogenization. In the strict sense, it can also be seen as a way to protect the bacterial population from the invasion of new genetic elements into genomes.

Indeed, there is no described mechanism for protection of bacteria from virulent phages, similar to the mechanism of CRISPR. In this case, there may be situations where the death of one separate population of bacteria may be a more effective way to protect the entire population from the spread of genes that are incompatible with the survival of the bacteria. In this respect, it would be interesting to search among the known CRISPR strains for such options, which would respond to infection by certain temperate phages just as to infection by virulent lytic phages.

## 14. Future Therapeutic Phages: Different Directions

If phage therapy is destined to become an accepted and widely used medical procedure, its maintenance and development will depend entirely on the success of the constant expansion of the collection of therapeutic bacteriophages with lytic activities against newly arising phage resistance of bacterial populations. Apparently, there will be the need for permanent development and modification of genetically engineered phages with required properties. There are possible several different approaches to reach this purpose. For instance, such unified phages could be engineered on the basis of certain existing species with an attempt to create a unified assembly of phages having in their genomes individual blocks of genes which can improve the efficiency of infection and lysis of different bacterial species. It is known that in some cases of removing genes of phage, genome can improve the phage growth, as in the case of *P. aeruginosa *phage F116 [136]. There are other examples of loss of a substantial part of the genome *P. aeruginosa *phages without sacrificing growth efficiency.

Temperate phage SM is capable of growing in P. aeruginosa PAO1 cells with addition insertion in its intentionally deleted shortened genome up to 12 kb DNA insertion [137]. Even more striking is the difference in the amount of phage DNAs in phages phiKZ and EL (NCBI access number NC_007623). Capsids of these phages have the same size, but their genomes show a great difference (280,334 bp for phiKZ and 211,216 bp for EL). Thus, the redundant capacity of DNA to be packed in an EL capsid is not less than 69 kb (!).

One of the possible areas of work in the creation of modified phages would be affording different phage species with the ability to recognize the type IV pili of different bacterial species. In this regard, there is a promising group of species showing relations with phage F116, whose genome is present in the cell as plasmids and that use pili of IV type for adsorption.

In phage therapeutic mixtures, different phages of the same species are often used, with different activities against various clinical isolates of *P. aeruginosa* [108]. It would be interesting to investigate the possibility of combining their specific features within a single phage genome. This will also help to start the work on unification of therapeutic phages.

Another possible trend for creating unified phages may be related to a deeper investigation of the origin of similar genes in genomes of phages active against bacteria of different species as *Salmonella enterica *and *Pseudomonas aeruginosa*, and possible mutual channels for migration. In relation to the hypothetical work on construction of artificial bacteriophages, looking for the elaboration of their industrial hosts will be required.

We consider that some principal changes related to the further development and application of therapy with live bacteriophages should be accomplished in the near future, as long as society has a high level of confidence in their use.
